# Structure transition of a C_60_ monolayer on the Bi(111) surface

**DOI:** 10.1039/d1ra00900a

**Published:** 2021-04-15

**Authors:** Ya-Ru Wang, Min-Long Tao, Ma Chao-Ke, Zi-Long Wang, Da-Xiao Yang, Ming-Xia Shi, Kai Sun, Ji-Yong Yang, Jun-Zhong Wang

**Affiliations:** School of Physical Science and Technology, Southwest University Chongqing China taotaole@swu.edu.cn jzwangcn@swu.edu.cn

## Abstract

The interfacial structures of C_60_ molecules adsorbed on solid surfaces are essential for a wide range of scientific and technological processes in carbon-based nanodevices. Here, we report structural transitions of the C_60_ monolayer on the Bi(111) surface studied *via* low-temperature scanning tunneling microscopy (STM). With an increase in temperature, the structure of the C_60_ monolayer transforms from local-order structures to a (√93 × √93) R20° superstructure, and then to a (11 × 11) R0° superstructure. Moreover, the individual C_60_ molecules in different superstructures have different orientations. C_60_ molecules adopt the 6 : 6 C–C bond and 5 : 6 C–C bond facing-up, mixed orientations, and hexagon facing-up in the local-order structure, (√93 × √93) R20°, and (11 × 11) R0° superstructure, respectively. These results shed important light on the growth mechanism of C_60_ molecules on solid surfaces.

## Abstract 2

With the increase in temperature, the structure of the C_60_ monolayer on the Bi(111) substrate transforms from local-order structures to a (√93 × √93) R20° superstructure, and then to a (11 × 11) R0° superstructure.

## Graphical abstract

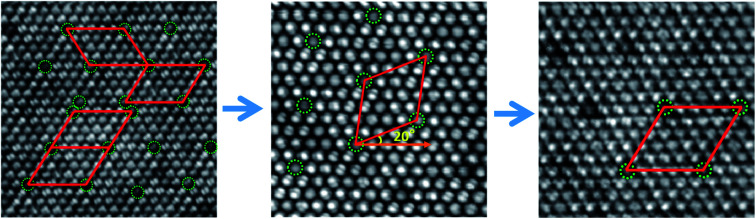

## Introduction

C_60_ molecule, as a prototypical fullerene molecule, has attracted widespread attention due to its potential in endohedral fullerenes,^1^ photovoltaic devices,^2^ peapod nanotubes,^3^ and single-molecule transistors.^4^ A C_60_ monolayer grown on solid surfaces is critical for understanding and controlling the interfacial properties of fullerene-derived electronic and photovoltaic devices.^5,6^ STM studies demonstrated that the C_60_ monolayer on the solid surface exhibit a variety of lattice orientations such as the “in phase” (2√3 × 2√3) R30°,^7–13^, (7 × 7) R0°^13^ and (√589 × √589) R14.5°.^13–15^ The individual molecules of fullerene and fulleride within a single domain display different orientations. In the complex orientational ordering (7 × 7) R0° structure, a 7-molecule C_60_ cluster consists of a central molecule sitting atop of a gold atom and six tilted surrounding molecules.^10^ In the unit cell of the (√589 × √589) R14.5° structure, 49 C_60_ molecules adopt 11 different orientations.^14^ In the (2√3 × 2√3) R30° structure, all C_60_ molecules are in the same orientation.^12,16^ The complex chiral motifs have been observed.^17^ In CsnC_60_ fulleride films, orientational ordering appears.^18^ Moreover, “bright” and “dim” molecules have been widely found in the C_60_ monolayer.^9–17^ However, the “dim” molecules in superstructures reported so far arrange irregularly.

The structure of C_60_ monolayers grown on the solid surface is not only related to C_60_ molecules themselves but also the substrate. In the past reports, there have been a large number of investigation on the C_60_ monolayer structures grown on numerous metals or semiconducting substrates, such as Ag,^7–9,19,20^ Au,^10–16,21,22^ Cu,^23–25^ graphene,^26,27^ Si,^28,29^ Ge,^30^ C_60_,^29^ or NaCl.^31^ However, few reports address the superstructure of C_60_ molecules adsorbed on semi-metal substrates. It is found that thin films of organic molecules grown on a semi-metallic Bi(111) surface shows a lot of interesting phenomena, such as the ordered crystalline layer with the standing-up orientation of pentacene molecules,^32^ the chiral self-assembly of rubrene molecules,^33^ structural transitions in different monolayers of cobalt phthalocyanine films,^34^ and the Moire' pattern in C_60_ thin films.^35^

In this study, we use Bi(111) as the substrate and studied the structure transition of the C_60_ monolayer. C_60_ molecules were deposited at 100 K form local-order structures. When the deposition temperature increased to room temperature, the local-order structures turn into a long-range ordered (√93 × √93) R20° superstructure. After annealing at 400 K, the ordered superstructure transforms into the (11 × 11) R0° superstructure. These superstructures are different from the structures of the C_60_ monolayer reported so far. Furthermore, the individual C_60_ molecules in the local-order structure, (√93 × √93) R20° and (11 × 11) R0° superstructure, show the 6 : 6 C–C bond and 5 : 6 C–C bond facing-up, mixed orientations, and hexagon facing-up, respectively. The 6 : 6 (5 : 6) C–C bond indicates the common side of two adjacent hexagons (pentagon and hexagon) in C_60_ molecules.

## Experimental

The experiments were conducted in an ultra-high vacuum low-temperature scanning tunneling microscope produced by Unisoku. The base pressure was kept at ∼1.2 × 10^−10^ Torr. An Si(111) substrate was continuously degassed at ∼870 K for 8 h with subsequent flashing to 1400 K for several seconds. The Bi(111) film was prepared by depositing 20 monolayers of bismuth atoms on a Si(111)-7 × 7 surface at room temperature with subsequent annealing at 400 K.^36^ C_60_ molecules were deposited onto the Bi(111) surface by heating the tantalum cell to 700 K. The growth rate of C_60_ molecules was about 0.4 monolayers per minute. All STM images were acquired with a tungsten tip in constant-current mode at liquid nitrogen temperature (78 K).

## Results and discussion

**Fig. 1:**
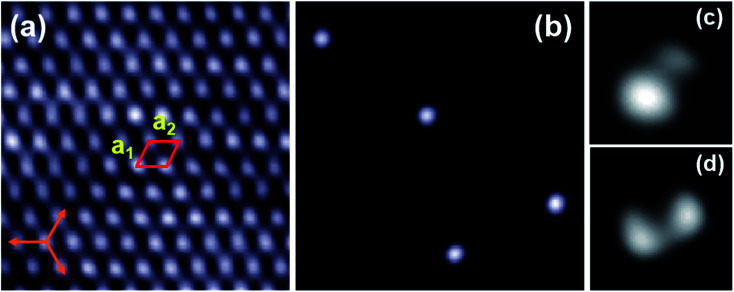
The initial stage of C_60_ molecules adsorbed on the Bi(111) surface. (a) Hexagonal lattices of the Bi(111) surface, 5 nm × 5 nm, −0.1 V. The unit cell is marked with a red box and the orange arrows indicate the directions of the Si(111) substrate. (b) Isolated C_60_ molecules adsorbed on Bi(111), 20 nm × 20 nm, 2.2 V. (c) STM image of an isolated C_60_ molecule with two asymmetrical lobes corresponding to the 5 : 6 C–C bond facing up, 1.3 nm × 1.3 nm, 400 mV. (d) STM image of an isolated C_60_ molecule with two symmetrical lobes corresponding to the 6 : 6 C–C bond facing up, 1.3 nm × 1.3 nm, 200 mV.

First, a small number of C_60_ molecules were deposited onto the Bi(111) surface when the substrate was maintained at 100 K. Fig. 1(a) shows the atomic-resolution image of the hexagonal lattices of the Bi(111) thin film. The lattice constants of the Bi(111) surface are measured to be *a*_1_ = *a*_2_ = 0.45 ± 0.02 nm, very close to the bulk value (*a* = 0.454 nm) in Bi crystals.^36^Fig. 1(b) shows the isolated C_60_ molecules on the Bi(111) surface presenting round protrusions. When reducing the bias voltage, the round protrusions are separated into two asymmetrical [Fig. 1(c)] or symmetrical [Fig. 1(d)] lobes, corresponding to the two different adsorption configurations, 5 : 6 C–C bond facing-up and 6 : 6 C–C bond facing-up, similar to C_60_ molecules on Au(111).^12^ This indicates that there are two stable adsorption orientations of isolated C_60_ molecules on the Bi(111) substrate, 6 : 6 C–C bond, and 5 : 6 C–C bond facing-up.

**Fig. 2:**
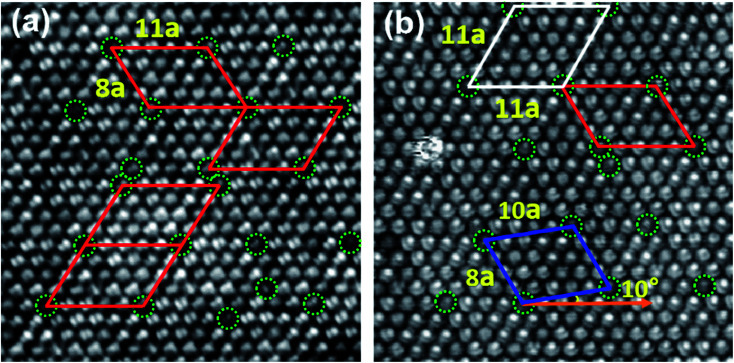
Local-order structure in the monolayer C_60_ grown at a low temperature (∼100 K). (a) Four unit cells of the (11 × 8) R0° superstructure appeared in the C_60_ monolayer, 20 nm × 20 nm, −1.2 V. The dim C_60_ molecules, located at the hollow position of Bi(111), are marked by the green dotted circles. (b) The mixture of three types of superstructures, 20 nm × 20 nm, −0.9 V. The red, white, and blue unit cells correspond to the superstructure (11 × 8) R0°, (11 × 11) R0°, and (10 × 8) R10°.

When the coverage increases, C_60_ molecules form the close-packed hexagonal structure, as shown in Fig. 2. We noticed that all the C_60_ molecules present a uniform height, except a few dim molecules (marked by green dotted circles). The brightness contrast in images stems from the different adsorption sites of C_60_ molecules. It is well known that metal surfaces do not behave as rigid templates for the chemisorption of C_60_ molecules, but may reconstruct substantially to accommodate the molecules.^37^ We speculate that Dim C_60_ molecules are located at the vacancies of the Bi(111) substrate, originating from the reconstruction of the Bi(111) surface, similar to C_60_ molecules on Au(111)^16^ and Cu(111).^38^

According to the arrangement of bright and dim molecules, we can see some local-order structures, though there is a lack of long-range ordering. In Fig. 2(a), there is an (11 × 8) R0° local-order structure (marked by red parallelogram). The lattice directions of (11 × 8) R0° are along with the directions of Bi(111), and the measured lattice constants are 5.00 ± 0.02 nm and 3.64 ± 0.02 nm, corresponding to 11 and 8 times of the lattice constant of the Bi(111) surface. The lattice directions of Bi(111) were obtained on the surface, which was not covered with C_60_ molecules. In another domain, shown in Fig. 2(b), the local-order structure is mixed with three types of structures, namely (11 × 8) R0° (red quadrilateral), (11 × 11) R0° (white quadrilateral), and (10 × 8) R10° (blue quadrilateral). In particular, we noticed that C_60_ molecules exhibit almost the same orientation in a single domain, and most of the individual C_60_ molecules in the local-order structure adopt two favorite orientations (6 : 6 C–C bond and 5 : 6 C–C bond facing up) as the isolated molecules on Bi(111). For example, most of the molecules shown in Fig. 2(a) present two symmetrical lobes, corresponding to C_60_ molecules with a 6 : 6 C–C bond facing up. However, in Fig. 2(b), the molecules present two asymmetric lobes, corresponding to the 5 : 6 C–C bond facing up. We suggest that the formation of a local-order structure is due to the low-temperature growth. Because of the low kinetic energy of C_60_ molecules at 100 K, molecular mobility is not high enough to form a long-range ordered superstructure. The C_60_ molecules adsorbed on Bi(111) adopt their preferred orientations (6 : 6 C–C bond and 5 : 6 C–C bond facing up), similar to the isolated molecules adsorbed on the substrate. This proves the strong molecule–substrate interaction in the local-order structure.

**Fig. 3:**
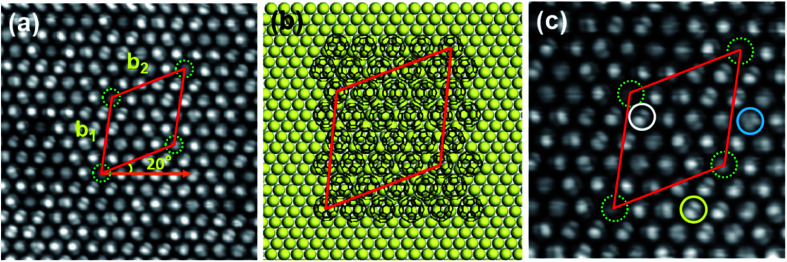
(a) The STM image of the (√93 × √93) R20° superstructure, 15 nm × 15 nm, −1.2 V. (b) Schematic model of the (√93 × √93) R20° superstructure. The yellow balls and black hollow balls represent Bi atoms and C_60_ molecules. (c) High-resolution STM image of the (√93 × √93) R20° superstructure, 10 nm × 10 nm, −1.0 V. The individual molecules exhibit different orientations, such as 5 : 6 C–C bond, 6 : 6 C–C bond, and hexagon facing up, marked by white, yellow, and blue solid circles, respectively.

To investigate the influence of temperature on the structure, we deposited C_60_ molecules on Bi(111) at room temperature. It is found that C_60_ molecules aggregate into a hexagonal structure, the same as C_60_ molecules in the local-order structure. However, the local-order structures, originating from the dim and bright molecules, turn into a long-range ordered (√93 × √93) R20° superstructure [Fig. 3(a)]. This superstructure is different from the structures of the C_60_ monolayer reported so far. There is a misorientation angle of 20° between the lattice directions of the C_60_ monolayer and the Bi(111) surface. The measured lattice constants of (√93 × √93) R20° are *b*_1_ = *b*_2_ = 4.38 ± 0.02 nm, agreeing well with √93 times the lattice constant of Bi(111) (0.45 nm). Fig. 3(b) shows the schematic of the (√93 × √93) R20° superstructure. Based on the lattice constant of the Bi(111) substrate, the lattice vectors of the (√93 × √93) R20° superstructure can be expressed as following matrixes:
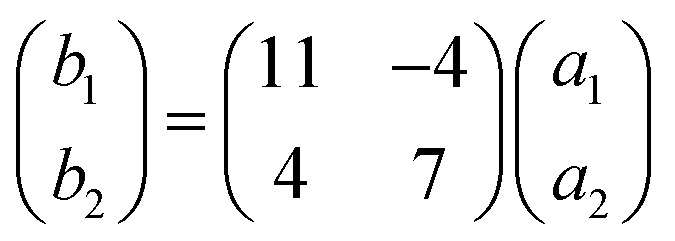


This ordered superstructure implies two things: first, the intermolecular interaction is getting stronger than that in the local-order structure prepared at low temperature (100 K). Second, the molecule–substrate interaction is also strong since the orientations of the C_60_ superstructure are commensurate with those of the substrate. Furthermore, we can clearly see that individual C_60_ molecules adopt various orientations, rather than the favorite orientations as C_60_ molecules in the local-order structure. As shown in the high-resolution STM image [Fig. 3(c)], C_60_ molecules in (√93 × √93) R20° present various shapes, such as two asymmetric lobes (white circle), two symmetrical lobes (yellow circle), and three lobes (blue circle), corresponding to the 5 : 6 C–C bond, 6 : 6 C–C bond, and hexagon facing up. The diversity of C_60_ molecular orientations is due to the enhancement of intermolecular interaction in the (√93 × √93) R20° superstructure. The intermolecular interaction enables C_60_ molecules to overcome the molecule–substrate interaction and adopt other orientations, and then make the (√93 × √93) R20° superstructure stable.

**Fig. 4:**
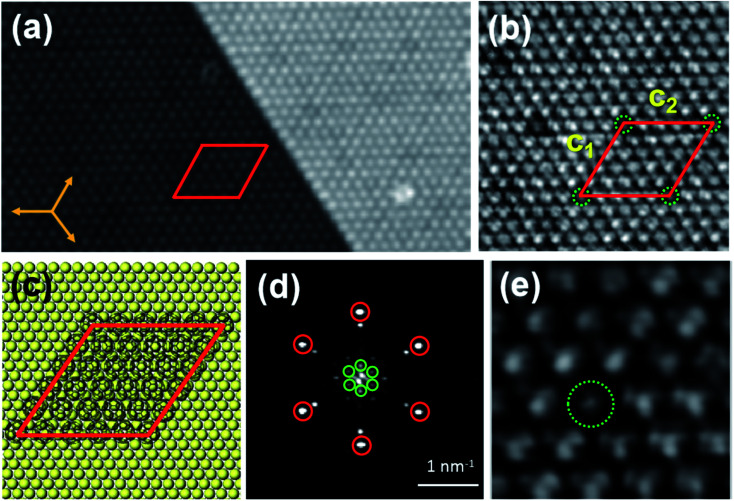
(a) The STM image of the (11 × 11) R0° superstructure corresponding to the Bi(111), 39 nm × 21 nm, −1.5 V. (b) Close-up view of the (11 × 11) R0° superstructure, 14 nm × 14 nm, −0.7 V. (c) Schematic model of the (11 × 11) R0° superstructure with respect to the Bi(111) lattices. (d) FFT of the image (a). The spots marked by red circles correspond to the C_60_ hexagonal lattices, while the spots marked by the green circles represent the (11 × 11) R0° superstructure. (e) STM image with a sub-molecular resolution of the superstructure, 5 nm × 5 nm, −0.7 V.

When annealed at 400 K for about 20 min, C_60_ molecules still revealed a hexagonal lattice, while the superstructure transformed from (√93 × √93) R20° into (11 × 11) R0° superstructure [Fig. 4(a)], indicating that (11 × 11) R0° is more stable than (√93 × √93) R20°. The lattice directions of (11 × 11) R0° are along the directions of the Bi(111) substrate, and the lattice constants are *c*_1_ = *c*_2_ = 5.00 ± 0.02 nm [Fig. 4(b)], corresponding to 11 times of the lattice constant of Bi(111). Fig. 4(d) is the fast Fourier transform (FFT) image of the (11 × 11) R0° superstructure, where the spots marked by red and green circles correspond to C_60_ hexagonal lattices and the (11 × 11) R0° superstructure. In the FFT image, the spots of the superstructure are clearly visible, though they are dimmer than the spots of C_60_ hexagonal lattices, implying that the (11 × 11) R0° superstructure has long-range order. The schematic model of (11 × 11) R0° is shown in Fig. 4(c). From STM images, the (11 × 11) R0° superstructure seems to have the same structure as the reported structure attributed to a Moire' pattern in ref. 36. However, in our experiment, the (11 × 11) R0° superstructure is transformed from the (√93 × √93) R20° superstructure and have no relationship with the Moire' pattern. From the close-up view of the (11 × 11) R0° superstructure in Fig. 4(e), it is found that all the C_60_ molecules reveal a unified three-lobe structure, corresponding to the hexagon facing up, different from favorite orientations in the local-order structure and mixed orientations in (√93 × √93) R20°. With an increase in temperature, the superstructure of the C_60_ monolayer changes from local order to long-range order and C_60_ molecules are re-orientated. This is because the thermal diffusivities of C_60_ molecules and Bi atoms increase with the increase in temperature, which is conducive to the formation of a more orderly and stable superstructure.

## Conclusions

In summary, the structure of C_60_ molecules on Bi(111) changes with temperature variation. When deposited on the Bi(111) surface at 100 K, C_60_ molecules form local-order structures, and the molecules in local-order structures adopt their favorite orientations. As the deposition temperature increases to room temperature, the local-order structures turn into a long-range ordered (√93 × √93) R20° superstructure. The orientations of C_60_ molecules in (√93 × √93) R20° superstructures are diverse. After annealing at 400 K for about 20 min, the C_60_ film exhibits a (11 × 11) R0° superstructure, and all C_60_ molecules in this superstructure take the unified orientation, hexagon facing-up. The appearance of numerous superstructures and the molecular orientations in superstructures is due to the change in the thermal diffusivity of C_60_ molecules and Bi atoms at different temperatures.

## Conflicts of interest

There are no conflicts to declare.

## Supplementary Material
